# Extra-laryngeal complications of suspension laryngoscopy

**DOI:** 10.1016/S1808-8694(15)31167-8

**Published:** 2015-10-19

**Authors:** Marco Antonio dos Anjos Corvo, Alessandra Inacio, Marina Bacal de Campos Mello, Cláudia Alessandra Eckley, André de Campos Duprat

**Affiliations:** 1 MD. Otorhinolaryngologist and graduate student in Laryngology - Faculdade de Ciências Médicas da Santa Casa de São Paulo, Attending Otorhinolaryngologist - Otorhinolaryngology Department - Irmandade da Santa Casa de Misericórdia de São Paulo; 2 MD. Otorhinolaryngologist - Otorhinolaryngology Department - Irmandade da Santa Casa de Misericórdia de São Paulo; 3 6th year Medical Student - Faculdade de Ciências Médicas da Santa Casa de São Paulo; 4 PhD. Assistant Professor - Otorhinolaryngology Department - Irmandade da Santa Casa de Misericórdia de São Paulo; 5 PhD. Assistant Professor - Otorhinolaryngology Department - Irmandade da Santa Casa de Misericórdia de São Paulo; Departamento de Otorrinolaringologia da Santa Casa de Misericórdia de São Paulo

**Keywords:** complications, laryngoscopy

## Abstract

Although suspension laryngoscopy is routinely used in laryngeal surgery, there are only few studies on the complications of this procedure.

**Aim:**

to evaluate the complications outside the larynx following suspension laryngoscopy and analyze their relation with surgery duration.

**Materials and Methods:**

Thirty-seven procedures were prospectively analyzed for intervention-related complications. The study included patient preoperative and postoperative assessment, focusing on dental, mucosal and nerve status (hypoglossal and lingual nerves).

**Results:**

Most procedures (27/37) were associated to some kind of complication, and mucosal injuries were the most common; temporary nerve lesions were observed in five cases and dental injuries in one case. Statistic significance was found between surgery duration and mucosal injury (lesions smaller than 1 centimeter), showing that longer procedure pose higher risks for these complications.

**Conclusion:**

These findings suggest that suspension laryngoscopy is frequently associated with complications outside the larynx. Although these injuries represent a low risk of significant morbidity, they can be avoided if more accurate techniques are used.

## INTRODUCTION

In 1912, Gustav Killian devised the suspension laryngoscopy (SL)^1^, ^2^, ^3^, bringing about a major progress to laryngeal procedures, allowing the surgeon to use both hands during procedures, facilitating the removal of soft tissue during surgical dissections. It is certain that such event brought about an exciting development to the field of laryngology, allowing or facilitating diagnostic and therapeutic approaches to laryngeal problems so far unknown.^1^ Despite its routine use today, there are very few studies in the literature dealing with possible complications to this procedure. Most of them are restricted to doing a retrospective analysis of the complications, without any preoperative analysis^4^. Even then, literature shows that minor complications such as dental and mucosal lesions (mild bleeding, bruises, blunt or cutting wounds) happen in 9.1% to 31% of the patients who undergo direct laryngoscopy.^5^, ^6^, ^7^, ^8^ Major complications which require hospitalization, such as pneumothorax, cardiovascular complications and intense bleedings have also been described, at an incidence varying between 0.4% to 19.5%, depending on the study.^5^, ^6^, ^7^, ^8^

Although rare, sensitive-motor lesions are also described in the literature as possible complications associated with suspension laryngoscopy. Gaut9 describes an injury to the lingual nerve during a SL of a 37-year-old man, after he complained of paresthesia and taste sense reduction on the right antero-lateral portion of his tongue. Cinar^10^ reports cases in which there were changes in the hypoglossal nerve after SL, detected by means of a tongue electromyography with a needle. Klussmann^4^ also detected function disorders in these two nerves stemming from suspension laryngoscopy in his recent study of 2002, confirming the occurence of such complication.

The goal of this present prospective study was to determine the frequency of extra laryngeal complications after suspension laryngoscopy, matching the presence of lesions with procedure duration.

## MATERIALS AND METHODS

We prospectively assessed all the patients submitted to suspension laryngoscopy between March and December of 2005, from an Otorhinolaryngology department of a tertiary University Hospital.

The patients were assessed in the pre-anesthetic period in order to rule out anatomical or functional lesions they had before the procedure, by means of a specific protocol created for this investigation. Such assessment included an oral cavity examination, paying special attention to:

• General teeth status
• Gum
• Lips
• Lining mucosa, movement, general - and specific tongue sensitivity
• Soft palate (including the uvula) and hard palate
• Oropharynx
• Mouth floor

The general sensitivity test was performed by the wooden tongue depressor stimulus against the right and left surface of the tongue, while the test of taste sensitivity was performed by placing small portions of sugar and salt on the tip and the lateral face of the tongue (right and left sides). Between each different taste stimulus, the patient cleaned his mouth with water in order to remove residues of the previous stimulation.

We decided to differentiate the uvula lesions from those found in the remaining soft palate in order to check for differences in the complication rates of these two regions.

After surgery, a new evaluation was carried out in order to identify early on the alterations stemming from suspension laryngoscopy. Those patients with sensitive-motor injuries were followed up until symptoms remission, for a maximum period of three months.

Regarding surgery duration, we divided the cases in three groups:

• Group A - surgery took less than 30 minutes
• Group B - surgery lasted between 30 and 60 minutes
• Group C - surgery took more than 60 minutes

Moreover, we also recorded the type of laryngeal disorder and the procedure performed. We used Holinger or Kleinsasser suspension laryngoscopes, which fit the larynx size of each patient.

### Inclusion criteria

We included those patients above 18 years of age, referred to direct suspension laryngoscopy for laryngeal microsurgery - for whatever the indication, who agreed to participate in the study and for that they signed an informed consent.

### Exclusion criteria

We excluded those patients below 18 years of age because of the technical difficulties to apply the research protocol in children and adolescents. We also excluded those patients in whom we could not perform direct laryngoscopy due to individual anatomical difficulties.

This study was approved by the Ethics in Research Committee of our institution, under protocol # 120905.

## RESULTS

We studied 36 consecutive patients who matched inclusion and exclusion criteria, and one of these patients underwent two different procedures at the time (in total we had 37 suspension laryngoscopies). We had 24 males and 12 females in our sample, with mean age of 48 years (varying between 18 and 80 years of age).

Preoperative diagnostic hypothesis of these cases were: neoplasia suspicion (14 cases), polyp (8 cases), Reinke's edema (5 cases), vocal fold cyst (4 cases), laryngeal papilloma (4 cases) and vocal fold groove (2 cases).

As to surgery time, 14 procedures lasted less than 30 minutes (Group A), 16 lasted between 30 and 60 minutes (Group B) and in only 7 patients, the surgery time went beyond 60 minutes (Group C).

The patients evolved without complications inherent to suspension laryngoscopy in 10 of the 37 procedures. In the remaining 27 procedures, the complications were didactically grouped in dental lesions, neurological lesions and injuries to the oral cavity.

One patient lost his tooth because of the SL. Five patients had neurological injuries. One of them had total alteration of tongue movement (tongue tip twisted to the right side), together with tongue paresthesia and reduction in right side taste sensation, with improvement after 3 months. Two patients had a reduction in their taste sense in half the tongue, one patient had tongue movement alteration associated with tongue paresthesia and taste impairment complaints, one patient had tongue movement impairment only and one had paresthesia alone in part of his tongue, and all these symptoms improved before sixty days of postoperative.

In absolute figures, we found 48 injuries in the oral cavity of the 36 patients studied. They were further organized in hematoma (21 cases), soft tissue edema (17 cases) and cuts (10 cases). These lesions were located in different parts of the mouth, hematomas were more frequently found in the soft tissue, cuts prevailed in the gum and lips, and soft tissue edema was more frequently found in the uvula.

All the labial and oral mucosa lesions had restitutium ad integrum healing, and were no longer seen in the first week of postoperative.

## DISCUSSION

In this study, we tried to use surgery time as an indicator of the time during which the suspension laryngoscope remained in the oral cavity. This parameter also informed us about the individual surgical difficulties related to the procedures, and we expected this factor to be related to a larger number of complications.

In fact, when we analyzed the data, we see that in Group A (less than 30 minutes of surgery) all patients evolved without complications detected, showing that there is a relationship trend between shorter procedures and the lack of complications. Thus, all procedures lasting for more than 60 minutes had at least one extra laryngeal complication related to SL, taking us back to that thought that the longer the surgery, the higher is the likelihood of complications. Through the Statistical Package for Social Sciences, version 13.0 software, the tests applied showed that there was indeed a relationship between surgery duration and oral mucosa injuries smaller than 1 centimeter (“p” − 0.045), allow us to state that the longer the procedure, the higher the number of such lesion in the oral mucosa.

We did not find in the literature data that correlate these two variables, or data that corroborate the results attained, thus yielding great importance to this preliminary study and reinforcing the need to continue with the study hereby presented.

Below we present some remarks about each one of the complications identified in the study:

### Dental injuries

Teeth injuries are known to happen in SL, and dental protection is widely recommended for such procedure.^11^ We know that prominent teeth and the need for serial endoscopic approaches increase the risk of dental injuries. Other things that increase this risk are dental cavities, periodontal disease, dental fillings and partially fixed dentures.^11^ In his study about the complications of suspension laryngoscopy, Klussmann4 advocated a preoperative dental assessment for all patients who would undergo the procedure, and found SL-related dental trauma in 92% of the patients with deep periodontal disease detected prior to surgery. He also found that no procedure-related dental complication was seen in patients with healthy teeth during the preoperative assessment, and these relations were statistically significant.

In the present investigation, the only case of SL-related dental trauma was detected in a patient with poor dental hygiene, corroborating Klussmann's previous data4. Therefore, preoperative dental assessment is mandatory in cases of dental frailty, in order to adopt more effective teeth protection measures against such complications, with proper teeth protection, and greater attention as one inserts and removes the laryngoscope from the oral cavity.

### Neurological injuries

The lingual nerve is responsible for the ipsilateral sensitive innervation of the tongue, inferior gum and mouth floor, causing loco-regional hyposthesia or paresthesia if damaged. Moreover, it is responsible for taste in the anterior two-thirds of the tongue through the corda tympani nerve (facial nerve branch) and for the innervation of the submandibular gland through parasympathetic fibers.^9^ Anatomically, the lingual nerve emerges from the posterior branch of the mandibular nerve in the pterigopalatine fossa, it meets the corda tympani (with facial nerve fibers) and goes downwards, between the medial and lateral pterigoid muscle and, afterwards, it passes between the medial pterigoid muscle and the mandible. Then, the nerve crosses over the hyoglossal muscle as it originates the terminal branches deep in the tongue.^12^

There are very few studies about intra-oral complications of suspension laryngoscopy, and they are all limited to assessing lingual nerve integrity, evaluating its function in providing general sensitivity to the tongue dorsum. In our investigation, we tried to add to this assessment by means of a taste stimulus based on salt and sugar in small amounts, thus increasing the investigation's sensitivity for potential injuries to the nerve fibers that could go unnoticed if only the general sensitive function was assessed.

Many lingual nerve injury mechanisms have already been proposed in the literature in order to explain how the trauma energy potentially present in this procedure is transmitted to the nerve fibers. The injury may happen:^13^,^14^

• between the pterigoid muscles - the maneuvers used to bring the mandible forward and mandible angle compression - used to keep the airway open - are described as cause of nerve injury by nerve compression between the lateral and medial pterigoid muscles.^15^,^16^
• between the medial pterigoid and the mandible - muscle contraction compresses the nerve against a hard bony surface (mandible).
• between the mandible's medial face and the posterior portion of the tongue - this part of the nerve may be very superficial and thus, more prone to injuries by the laryngoscope blade during forced laryngoscopy.^9^
• as it crosses the hyoglossal muscle, cricoid cartilage compression prevents the hyoid muscle to move forward together with the tongue during laryngoscopy, causing a stretching and straining force on the nerve.

Injuries to the hypoglossal nerve may be equally related to intraoral procedures, such as SL. We know that this nerve emerges from the medulla oblongata and has its apparent origin in the cranial vault through the hypoglossal canal, moving downwards between the great veins of the neck all the way to the mandible angle. It then moves below the digastric muscle, it penetrates the oral cavity and innervates all the intrinsic and extrinsic tongue muscles, except the palatoglossal muscle. Injuries to this nerve impairs tongue motor function, making it deviate to the injury side when the inferior motor nerve is injured.^10^ Dziewas and Lüdemann^17^ described a series of hypoglossal nerve injuries in 2002, reporting 6 cases related to prior suspension laryngoscopy, and 5 out of the 6 reported cases improved.

As it happens to the lingual nerve, many are the hypothesis that tries to explain the physiopathological mechanisms involved in hypoglossal nerve injuries after oropharynx approaches:

• anterior tongue shift during laryngoscopy causing strain on the nerve^16^
• proximity to the hyoid bone makes the nerve vulnerable to the compression forces between the hyoid and the laryngoscope blade^18^
• hypoglossal nerve compression between the hyoid bone and the calcified stylohyoid ligament^19^,^20^

Some authors also advocate that the hypoglossal nerve, classically a motor nerve only, may have an afferent component that innervates the tongue, also causing sensitivity alterations when injured.^12^,^21^

The rarity of the nerve injuries presented suggest that the cause of such events be multifactorial, with anatomical and technical factors participating equally.^10^,^12^ Nonetheless, a careful attention should be exercised when placing the laryngoscope, and reducing the pressure it causes on the tongue base may help reduce even further the incidence of injuries to the nerves mentioned.^10^

In the present investigation, there were five patients with neurological complaints. One of them had a simultaneous deficit in the lingual and hypoglossal nerves, complaining of paresthesia and taste reduction on the right side of the tongue associated with tongue tip deviation towards the injured side. In such a case, one may infer that the injury forces were concentrated on the right hemi tongue because it caused a functional deficit in both nerves of the same side. The literature has reports of concurrent hypoglossal and lingual nerve injuries, however stemming from intubation and direct laryngoscopy.^19^ We did not find reports of these neural injuries stemming from SL.

In this case in particular, the patient improved in his tongue movement within thirty days, while the paresthesia and the taste disorder regressed progressively until the 60th postoperative day, indicating that it was a temporary nerve injury. We are still uncertain about the reason why there was no simultaneous function improvement in both nerves; it may be related to the anatomical trajectory of both, with a greater frailty of the sensitive fibers, or possibly with a less healing capacity of the lingual nerve.

Two patients had isolate functional deficit in the lingual nerve that was perceived by taste feeling reduction only, curiously without any complaint as to general sensitivity (hypoesthesia or paresthesia). The idea we had is that only the corda tympani nerve fibers were injured during the procedure. We did not find in the literature any other study investigating lingual nerve function deficit by stimulating the corda tympani nerve fibers, which are part of it, as aforementioned^4^, ^5^, ^6^, ^7^, ^8^, ^9^, ^10^. Taste evaluation was carried out as a means to broaden or confirm SL-related lingual nerve injury. If the taste study had not been made, it may be that such complication would not have been detected, since the patient himself had not noticed such dysfunction. We assume the lack of dysfunction awareness comes from the integrity of receptor fibers in the remaining oral mucosa.

As to nerve function recovery, both reported significant and progressive symptoms improvement after 7 days of progression.

Finally, the two remaining patients who had some neurological injury after suspension laryngoscopy evolved with tongue movement disorders and consequent lingual paresis. Both evolved well with symptoms regression after the seventh postoperative day.

As to the physiopathology of nerve injuries, the harmful effects of straining forces on the nerve physiology have been broadly studied for peripheral nerves. Schemia caused by blood flow interruption to the nerve is seen as a partial reason for this injury.^9^,^22^ The same harmful effects have been reported in the literature for the lingual and hypoglossal nerves, which were the goals of our evaluation.^4^ As to nerve function recovery, there are reports of improvement after four weeks in average for lingual nerve injuries and after 8 weeks for hypoglossal nerve injuries.^4^ Therefore, our findings in this study are in accordance with the data present in the literature.

### Oral cavity lining injuries

All injuries detected on the oral cavity surface are of spontaneous remission, becoming mild sequelae in the endoscopic procedure.^4^ The fact that these were the ones most commonly found prove the procedure relatively safe, because it did not show life threatening complications.

Understanding the mechanisms involved in the origin of these mucosal injuries may help in the development of methods to prevent them, and may be relevant to SL practice.^4^ In fact, one may consider that such complications are determined by placing the laryngoscope blade inside the oral cavity, and such maneuver is repeated until one can achieve proper surgical field exposure before using the microscope.

Another factor to be considered is the experience and training of the physician in charge of the procedure. This study was carried out in a tertiary University Hospital and third year otorhinolaryngology residents carried out the surgical procedures. This could explain the greater incidence of oral cavity injuries, since learning to place the rigid laryngoscope requires multiple attempts until one manages to properly expose the larynx. The surgeons concentrate mainly in the larynx and underestimate the possible complications associated with access. Care must be taken in choosing laryngoscope size and type, and its placement technique, in order to avoid or mitigate the aforementioned complications.

## CONCLUSIONS

1. Oral cavity lining injuries were the most frequent complications stemming from suspension laryngoscopy in this investigation, with five cases of nerve injuries and one tooth injury.
2. In the present investigation, we could see that longer surgeries are associated with more surface injuries smaller than one centimeter in diameter. A continuation of the present study is necessary in order to check for other possible relations between time and associated injuries.
3. These findings show that suspension laryngoscopy in itself is an innocuous procedure, and it may reduce frequent complications. It does not cause major harm to patients and such damages are avoidable as long as better techniques are used, safety measures are taken and care is exercised in the beginning of the procedure which is the very placement of the suspension laryngoscope.
